# Detection of rare carbapenemases in Enterobacterales—comparison of two colorimetric and three CIM-based carbapenemase assays

**DOI:** 10.1128/spectrum.03015-23

**Published:** 2024-01-17

**Authors:** Lukas Schaffarczyk, Janina Noster, Yvonne Stelzer, Janko Sattler, Sören Gatermann, Axel Hamprecht

**Affiliations:** 1 Klinikum Oldenburg, Institute of Medical Microbiology and Virology, Oldenburg, Germany; 2 Carl von Ossietzky University Oldenburg, Institute of Medical Microbiology and Virology, Oldenburg, Germany; 3 Institute for Medical Microbiology, Immunology and Hygiene, University Hospital of Cologne, Cologne, Germany; 4 Department of Medical Microbiology, Ruhr University Bochum, Bochum, Germany; Emory University School of Medicine, Atlanta, Georgia, USA

**Keywords:** carbapenemase, CPE, OXA-23, OXA-48, NDM, OXA-58, KPC, GIM, IMI, GES

## Abstract

Rapid and reliable detection of carbapenemase-producing Enterobacterales (CPE) is crucial for prompt treatment and infection control. Most assays target the primary four enzymes (KPC, OXA-48-like, VIM, and NDM), often missing less common variants (e.g., GES, IMI, OXA-23, and OXA-58). Therefore, assays based on the hydrolysis of carbapenems are recommended in addition to differentiation tests such as PCR or immunochromatographic assays. The aim of this study was to compare the currently Clinical and Laboratory Standards Institute (CLSI)-recommended tests mCIM (modified carbapenem inactivation method) and Carba NP with new colorimetric tests (NitroSpeed-Carba NP) and novel variations of the carbapenem inactivation method (CIM) such as simplified CIM (sCIM) or modified zinc-supplemented CIM (mzCIM). The challenge collection included 205 clinical isolates, 139 CPE vs 66 non-CPE. Among all 205 isolates, the sensitivity/specificity of mCIM was 81.3%/98.5%, Carba NP 76.3%/100%, NitroSpeed-Carba NP 86.3%/78.8%, sCIM 100%/94%, and mzCIM 97.8%/98.5%. For rare carbapenemases (*n* = 48), the sensitivity of mzCIM (98.3%) and sCIM (100%) was higher than that of mCIM (60.4%), Carba NP (50%), or NitroSpeed-Carba NP (70.2%). Most indeterminate results occurred for mCIM (14.4%), Carba NP (8.2%), and sCIM (6.3%). The detection of rare carbapenemases remains challenging with the currently recommended assays. The CIM-based tests demonstrated superior sensitivity, with sCIM and mzCIM outperforming the currently recommended mCIM and Carba NP, especially among isolates with weakly hydrolyzing carbapenemases (e.g., OXA-23 and OXA-58). Although colorimetric assays provide more rapid results, laboratories have to be aware of the low sensitivity for rare carbapenemases. Both sCIM and the new mzCIM performed well, are cost-effective, and can easily be implemented in any laboratory.

**IMPORTANCE:**

Detection of so-called rare carbapenemases (e.g., GES, IMI, OXA-23, and OXA-58) in Enterobacterales is challenging, and data on the performance of currently available assays are scarce. This study systematically assessed the performance of currently recommended and novel hydrolysis-based assays on a set of molecularly characterized isolates. It demonstrates that the currently recommended assays mCIM and Carba NP perform well on isolates producing common carbapenemases such as KPC, VIM, NDM, and OXA-48, but have only a moderate sensitivity in the detection of rare carbapenemases. In contrast, the newer CIM-based variants, sCIM and mzCIM, are equally capable of detecting frequent and uncommon carbapenemases. These assays could potentially help to improve our knowledge on the epidemiology of these “rare” enzymes.

## INTRODUCTION

The World Health Organization declared antimicrobial resistance as one of the ten most urgent threats to global health (1). Antibiotics as an achievement of modern medicine increasingly lose their power, and only the targeted and economical use of these drugs can counteract the spread of resistance. Carbapenem antibiotics are among the most valuable antibiotics against infections caused by Enterobacterales and other gram-negative bacilli.

Resistance to carbapenems can be caused not only by the loss of cell membrane permeability in combination with hyperproduction of AmpC β-lactamases or extended spectrum β-lactamases (ESBL), but also by the expression of carbapenemases (2–5).

Carbapenemases are able to hydrolyze and thereby inactivate carbapenems and most of the other β-lactam antibiotics. In order to prevent the spread of carbapenemase-producing Enterobacterales (CPE) and to treat patients adequately, susceptibility testing, as well as rapid and reliable identification of pathogens with carbapenemases, is of utmost importance (2). Most carbapenemase tests for Enterobacterales target only the four main enzymes (KPC, OXA-48-like, VIM, and NDM), and less common carbapenemases (GES, IMI, GIM, OXA-23, and OXA-58) are often not detected (6–9). This includes most commercially available genotypic assays (e.g., PCR assays and loop-mediated isothermal amplification [LAMP]), which do not target carbapenemases such as GIM, GES, or OXA-23/OXA-58 (5). Additionally, these tests are expensive and may not be available in all laboratories. Therefore, phenotypic assays that can be performed in any laboratory at low cost are indispensable for the routine laboratory.

In order to identify significantly rarer carbapenemases, assays based on carbapenem hydrolysis (activity tests) are recommended by the National Antimicrobial Susceptibility Committee Germany (NAC), European Committee on Antimicrobial Susceptibility testing (EUCAST), and Clinical and Laboratory Standards Institute (CLSI), in addition to differentiation tests such as PCR or immunochromatographic assays (10–13).

Currently, many different hydrolysis-based assays are available, including colorimetric tests like Carba NP, NitroSpeed-Carba NP, and different variants of the carbapenem inactivation method (CIM), e.g., modified CIM (mCIM), zinc-supplemented CIM (zCIM), or simplified CIM (sCIM) (5, 12, 14).

Although the different assays have been assessed mostly on isolates producing the primary four carbapenemases (KPC, OXA-48-like, VIM, and NDM), little data are available on the performance of these tests for the detection of rare carbapenemases—the indication where they are most useful.

The aim of this study was to compare currently recommended methods for carbapenem detection (mCIM and Carba NP) with a new colorimetric test (NitroSpeed-Carba NP) and the recently introduced CIM-based methods, sCIM and mzCIM, especially with regard to the detection of rare carbapenemases.

## MATERIALS AND METHODS

### Bacterial isolates

All clinical isolates were obtained from the institutes of medical microbiology at the university hospitals of Oldenburg and Cologne, as well as from the German National Reference Centre for multidrug-resistant Gram-negative bacteria at the University of Bochum (15–17). All CPE isolates were analyzed by whole genome sequencing using short-read technology (Illumina, San Diego, CA, USA), as previously reported (18). A total of 205 clinical Enterobacterales isolates were used to assess the tests*;* these included *Klebsiella pneumoniae/variicola* (*n* = 50), *Proteus mirabilis* (*n* = 32), *Enterobacter cloacae complex* (*n* = 37), *Escherichia coli* (*n = 30*), *Citrobacter freundii* (*n* = 17), *Serratia marcescens* (*n* = 13), and others (*n* = 26). Of these, 139 isolates were CPE and 66 were carbapenemase-negative, including ESBL- (*n* = 20) and AmpC-producers (*n* = 19) or a combination of both mechanisms (*n* = 16) (Table 2; Table S1). Most carbapenemase-negative control strains (58/66, 88%) were resistant at least to one of the carbapenems.

Isolates were grown overnight at 37°C on Columbia blood agar (BD, Heidelberg, Germany). Susceptibility testing was carried out by Vitek2 AST223 card (bioMérieux, Nürtingen, Germany) and gradient tests (Liofilchem, Roseto degli Abruzzi, Italy), minimal inhibitory concentrations were interpreted according to EUCAST 13.0 breakpoints. *E. coli* NCTC 13476 (IMP-1) and *K. pneumoniae* BAA 2814 (KPC-3) were used as quality control strains for the five carbapenemase assays. All tests were read by a person who was blinded to the molecular characterization results of the isolates.

### Modified carbapenem inactivation method (mCIM)

mCIM was performed as specified by the Clinical and Laboratory Standards Institute (19): a 1-µL loop full of bacteria was inoculated in 2 mL Brain Heart Infusion (BHI; BD, Heidelberg, Germany) and vortexed for 15 seconds. A 10-µg meropenem disc (Oxoid, Wesel, Germany) was added to the suspension and incubated for 4 hours at 35 ± 1°C in ambient air, before the disc was placed on a Mueller Hinton agar plate (MHA; Oxoid), previously inoculated with a susceptible *E. coli* indicator strain (ATCC 25922; 0.5 McFarland). The inhibition zone was measured after an 18-hour incubation at 35 ± 1°C in ambient air. Zone diameters of 6–15 mm or the presence of pinpoint colonies within a 16- to 18-mm zone were regarded as positive. Zone diameters of ≥19 mm were interpreted as negative, whereas 16–18 mm or a diameter of ≥19 mm with the presence of pinpoint colonies within the zone was considered indeterminate.

### Modified zinc-supplemented carbapenem inactivation method (mzCIM)

For this study, a modified version of the zinc-supplemented CIM was employed (7, 20). Briefly, two full 10-µL inoculation loops of bacterial colonies were suspended in 400-µL BHI supplemented with ZnSO_4_ (1.5-mM final concentration). A 10-µg meropenem disc was added to the suspension and incubated for 4 hours at 35 ± 1°C, before the disc was placed on a Mueller Hinton agar plate, previously inoculated with a susceptible *E. coli* indicator strain (ATCC 25922; 0.5 McFarland). The inhibition zone was measured after an 18-hour incubation at 35 ± 1°C. Thresholds were ≤18 mm for carbapenemase production, 19–20 mm was regarded as indeterminate, and ≥21 mm as negative. Satellite colonies in the inhibition zone were considered as positive.

### Simplified carbapenem inactivation method (sCIM)

The sCIM test was carried out as previously described (14). Briefly, imipenem discs were impregnated with the isolate to be tested, which were then placed upside down on a MHA plate previously inoculated with *E. coli* ATCC 25922. Plates were incubated at 35°C for 18 hours. Inhibition zones of 6–20 mm or colonies within a ≤22-mm zone were considered positive, an inhibition zone ≥26 mm as negative, and a zone of inhibition of 23–25 mm as indeterminate (14, 21).

### NitroSpeed-Carba NP test

The NitroSpeed-Carba NP test was conducted as described by Nordmann et al. (22) with slight modifications: the reaction volume was reduced from 175 to 87.5 µL, and only the tubes 1 and 2, out of five tubes, were used in this work, allowing the discrimination between carbapenemase producers and carbapenemase-negative isolates, whereas the additional reactions for the discrimination of different carbapenemase classes were omitted. The test was read after 15 minutes. If the color changed from yellow to red or reddish-yellow, the reaction was read as positive (Fig. S1). A color change in tube 1 indicates the presence of a β-lactamase, a color change in tube 2 indicates the presence of a carbapenemase.

### Carba NP

Carba NP test was carried out as previously described (23, 24), using 0.1% (vol/vol) Triton X-100 for bacterial lysis. The criteria established by CLSI were applied for the interpretation of results (19): the test was considered positive if the color changed from red to yellow, dark yellow, or light orange. Orange was interpreted as invalid, and red or red-orange as negative.

### Statistical analysis

The sensitivity and specificity of the tests were calculated using molecular characterization by whole genome sequencing as the reference. Indeterminate results were considered negative for the calculation of sensitivity and specificity. Additionally, the 95% confidence intervals and the Youden index were calculated. GraphPad Prism 8.1 was used for creating violin plots.

## RESULTS

In this study, two colorimetric and three carbapenem inactivation methods were assessed on a collection of 139 molecularly characterized CPE and 66 non-CPE. The aim of the study was to determine the performance of the currently recommended methods mCIM and Carba NP with recently developed assays (mzCIM, sCIM, NitroSpeed-Carba NP) to detect rare carbapenemases (e.g., GES, IMI, GIM or OXA-23, and OXA-58), which are not targeted by most commercial molecular and/or immunochromatographic tests.

### Performance of carbapenemase detection assays

The overall sensitivity of the five tests ranged from 76.3% (CI 69%–83%) for Carba NP to 100% (CI 97%–100%) for sCIM (Table 1).

**TABLE 1:** Performance of carbapenemase-activity tests for 139 CPE and 66 non-CPE Enterobacterales^*a*^

	Sensitivity % (95% CI)	Specificity % (95% CI)	Indeterminate %	Youden index
CIM-based assays				
Modified CIM (mCIM)	81.3 (74–87)	98.5 (92–100)	14.4	0.80
Modified zCIM (mzCIM)	97.8 (94–99)	98.5 (92–100)	0	0.96
Simplified CIM (sCIM)	100 (97–100)	94 (86–98)	6.3	0.93
Colorimetric assays				
Carba NP	76.3 (69–83)	100 (95–100)	8.2	0.76
NitroSpeed-Carba NP	86.3 (80–91)	78.8 (68–87)	–	0.65

^*a*^ For the calculation of sensitivity and specificity, indeterminate results were counted as negative.

**Fig 1:**
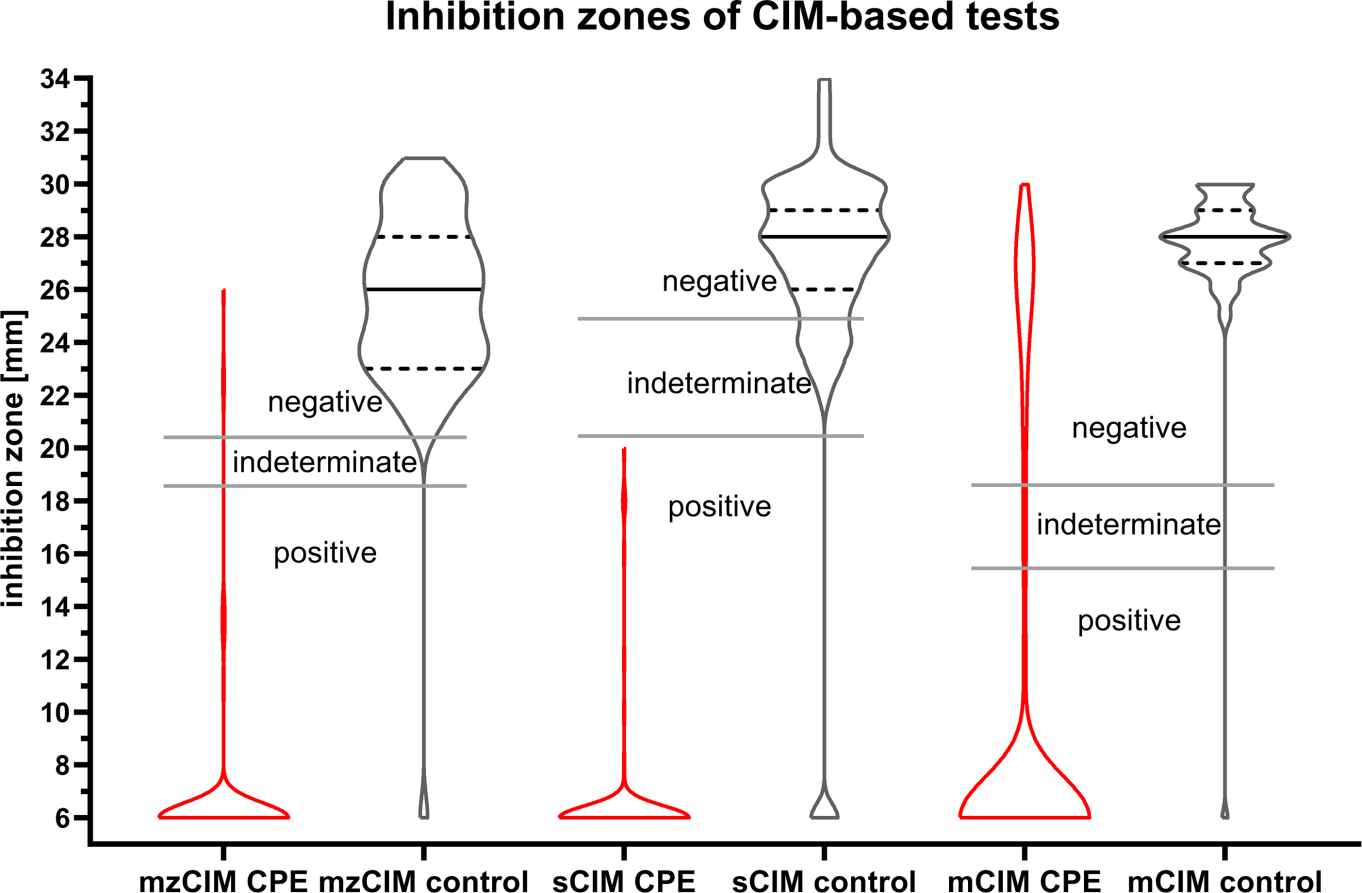
Violin plots of inhibition zones for all 205 Enterobacterales, stratified by carbapenemase production. CPE, carbapenemase-producing Enterobacterales; control, carbapenemase-negative Enterobacterales.

The new variants of the CIM-based tests (sCIM and mzCIM) demonstrated overall superior performance in comparison to mCIM and the colorimetric tests, with a sensitivity of 97.8% for mzCIM (CI 94%–99%) and 100% for sCIM (CI 97%–100%). Between the three CIM-based tests, a higher specificity of 98.5% (CI 92%–100%) was recorded for mCIM and mzCIM, compared to 94% (CI 86%–98%) for sCIM. For the colorimetric tests, a lower sensitivity was recorded (NitroSpeed-Carba NP 86.3%, Carba NP 76.3%). Carba NP was the only test that reached a specificity of 100% (CI 95%–100%). The Youden index was highest for mzCIM (0.96) and lowest for NitroSpeed-Carba NP (0.65). Carba NP, mCIM, and sCIM yielded the highest proportion of indeterminate results, with rates of 8.2%, 14.4%, and 6.3%, respectively (Table 1; Fig. 1).

### Class-specific performance of carbapenemase detection assay

The highest sensitivity for the four most common carbapenemases (NDM, VIM, OXA-48-like, and KPC) was recorded for sCIM (100%, CI 97%–100%) and mzCIM (98.9%, CI 95%–100%) (Table 3). Conversely, NitroSpeed-Carba NP demonstrated a sensitivity of 95.2% (CI 88%–98%), successfully detecting all VIM carbapenemases but missing 1/34 OXA-48-like carbapenemases (sensitivity 97.1%) and 3/16 NDM carbapenemases (sensitivity 81.3%).

Carbapenemases of Ambler Class A and B were detected by mzCIM and sCIM (sensitivity 100%, CI 95.3%–100%), whereas the performance of mCIM and the colorimetric tests was moderate. mCIM showed reduced sensitivity (93.8%) in detecting NDM and VIM-producing isolates (sensitivity 68.2%). NitroSpeed-Carba NP failed to detect some GES (sensitivity 66.7%), IMI (sensitivity 88.9%), and NDM (sensitivity 81.3%), whereas Carba NP showed weakness in the detection of VIM (sensitivity 95.5%), IMP (sensitivity 90.9%), as well as GES (sensitivity 0%) and IMI (sensitivity 77.8%), as previously reported (25, 26). The well-known problem of Carba NP tests in detecting OXA-244 was also evident with our set of isolates (sensitivity 28.6%) (Table 2) (17, 23, 26).

**TABLE 2:** Performance of the five assays for the detection of carbapenemase production^*a*^

	mCIM	mzCIM	sCIM	Carba NP	NitroSpeed-Carba NP
	Sensitivity (%) (CI)	Sensitivity (%) (CI)	Sensitivity (%) (CI)	Sensitivity (%) (CI)	Sensitivity (%) (CI)
All carbapenemases (*n* = 139)	81.3 (74–87)	97.8 (94–99)	100 (97–100)	76.3 (69–83)	86.3 (80–91)
Ambler Class A (*n* = 25)	92	100	100	79.2	87.5
KPC (*n* = 12)	100	100	100	100	100
GES (*n* = 4)	50	100	100	0	50
IMI (*n* = 9)	100	100	100	77.8	88.9
Ambler Class B (*n* = 53)	77.4	100	100	96.2	94.2
NDM (*n* = 16)	93.8	100	100	100	81.3
VIM (*n* = 22)	68.2	100	100	95.5	100
IMP (*n* = 12)	83.3	100	100	90.9	100
GIM (*n* = 3)	33.3	100	100	100	100
Ambler Class D (*n* = 54)	75.9	96.3	100	54.5	74.5
OXA-23 (*n* = 9)	66.7	100	100	0	22.2
OXA-48-like					
OXA-48 (*n* = 11)	100	100	100	100	90.9
OXA-162 (*n* = 5)	100	100	100	80	100
OXA-181 (*n* = 6)	100	100	100	83.3	100
OXA-232 (*n* = 2)	100	100	100	100	100
OXA-244 (*n* = 7)	100	85.7	100	28.6	100
OXA-245 (*n* = 2)	100	100	100	100	100
OXA-370 (*n* = 1)	100	100	100	100	100
OXA-58 (*n* = 11)	9.1	91	100	9.1	45.5
Double carbapenemases (*n* = 7)	100	100	100	100	100
NDM-1 + OXA-48 (*n* = 3)	100	100	100	100	100
NDM-1 + OXA-232 (*n* = 2)	100	100	100	100	100
NDM-5 + OXA-181 (*n* = 1)	100	100	100	100	100
KPC-2 + OXA-48 (*n* = 1)	100	100	100	100	100

^*a*^ CI, 95% confidence interval; mCIM, modified carbapenem inactivation method; mzCIM, modified zinc-supplemented carbapenem inactivation method; sCIM, simplified carbapenem inactivation method.

Similarly, the colorimetric tests did not perform well in recognizing Class D carbapenemases. The sensitivity for detecting OXA-23 (*P. mirabilis*) varied, with 0% for Carba NP, 22.2% for NitroSpeed-Carba NP, and 66.7% for mCIM, whereas both mzCIM and sCIM achieved 100%. OXA-58 was successfully detected by sCIM (sensitivity 100%) and mzCIM (sensitivity 91%), whereas NitroSpeed-Carba NP (sensitivity 45.5%), mCIM (sensitivity 9.1%), and Carba NP (sensitivity 9.1%) showed limited ability to detect these weakly hydrolyzing enzymes (Table S2).

NitroSpeed-Carba NP generated the most false-positive results (specificity 78.8%, CI 68–87%), most commonly in *C*. *freundii* and *Enterobacter cloacae complex* isolates producing AmpC β-lactamases (ACT or CMY) and predominantly elevated MICs for ertapenem and imipenem (Table S2).

## DISCUSSION

The currently recommended modified CIM (mCIM) test demonstrated robust sensitivity in initial studies (12, 27). However, subsequent investigations have revealed false-negative results, particularly in the detection of metallo-β-lactamases. Consequently, a number of different versions of the CIM-based tests have been described (7, 14, 21, 28, 29). Previous studies have shown that adding zinc sulphate can improve the detection of metallo-β-lactamases (zCIM), but some isolates producing GES, VIM, or IMI remained undetected (7, 15, 17). Therefore, zCIM was modified (mzCIM), including a higher inoculum, increased zinc concentration, and extended incubation time (4 hours), resulting in a higher sensitivity for Ambler Class A and B carbapenemases (100%, CI 95%–100%) and Class D carbapenemases (96.3%, CI 88%–99%) (20).

Presently, there is a scarcity of data regarding the performance of carbapenemase assays for OXA-23 and OXA-58 detection in Enterobacterales. OXA-23 and OXA-58 are primarily found in *Acinetobacter* spp. and rarely in Enterobacterales, with *Proteus* spp. being the predominant genus in the latter. Recently, it has been demonstrated that the detection of carbapenemases in *P. mirabilis* isolates is particularly difficult, despite carbapenemase production isolates usually having low carbapenem MICs. Additionally, phenotypic carbapenemase assays frequently give rise to false-negative results. Both sCIM and mzCIM have shown a higher sensitivity for detecting carbapenemases in *Proteus* spp. compared to mCIM (63% sensitivity) and Carba NP (30% sensitivity), especially in isolates producing OXA-23 and OXA-58 (20). For improved detection of carbapenemases in this challenging species, a diagnostic algorithm based on ticarcillin-clavulante, temocillin, and mzCIM has been developed, which has shown a sensitivity and specificity of 100% (20).

Additional data for OXA-23 and OXA-58 are only available for sCIM and Carba NP, but are mostly limited to *Acinetobacter* isolates. For both assays, positive results in OXA-23 and OXA-58 producing *Acinetobacter* isolates have been reported. Data on the performance of NitroSpeed-Carba NP in detecting OXA-23 and OXA-58 have not been available until now (14, 23).

For the identification of rare carbapenemases, modified CIM (mCIM) and the colorimetric tests have shown an overall lower performance compared to the new CIM tests-variations mzCIM and sCIM (Table 3). The mCIM and Carba NP tests, widely employed in numerous studies and endorsed by authoritative bodies such as CLSI and EUCAST for carbapenemase detection, have limitations in effectively detecting certain carbapenemases. These findings shed light on the need for further advancements in carbapenemase detection methodologies to address the challenges posed by these elusive enzymes (23, 26, 29, 30). In the present study, carbapenemases of the type GES-5, OXA-244, but also OXA-23 and OXA-58, in Enterobacterales were not sufficiently detected by Carba NP. Similarly, mCIM missed some NDM, VIM, GES, IMP, GIM, OXA-23, and OXA-58 producing Enterobacterales.

**TABLE 3:** Performance of five assays for detection of the main four and rare carbapenemases^*a*^

	mCIM		mzCIM		sCIM		Carba NP		NitroSpeed-Carba NP	
	Sens (CI)	Spec (CI)	Sens (CI)	Spec (CI)	Sens (CI)	Spec (CI)	Sens (CI)	Spec (CI)	Sens (CI)	Spec (CI)
All carbapenemases (*n* = 139)	81.3 (74–87)	98.5 (92–100)	97.8 (94–99)	98.5 (92–100)	100 (97–100)	94 (86–98)	76.3 (69–83)	100 (95–100)	86.3 (80–91)	78.8 (68–87)
Main four carbapenemases (*n* = 84)	90.5 (82–95)		99.2 (95–100)		100 (97–100)		90.5 (82–95)		95.2 (88–98)	
KPC (*n* = 12)	100		100		100		100		100	
NDM (*n* = 16)	93.8		100		100		100		81.3	
VIM (*n* = 22)	68.2		100		100		95.5		100	
OXA-48-like (*n* = 34)	100		97.1		100		79.4		97.1	
Rare carbapenemases (*n* = 48)	60.4 (46–73)		98.3 (94–100)		100 (97–100)		50 (36–64)		70.2 (56–81)	
GES (*n* = 4)	50		100		100		0		66.7	
IMI (*n* = 9)	100		100		100		77.8		88.9	
IMP (*n* = 12)	83.3		100		100		90.9		100	
GIM (*n* = 3)	33.3		100		100		100		100	
OXA-58 (*n* = 11)	9.1		91.0		100		9.1		45.5	
OXA-23 (*n* = 9)	66.7		100		100		0		22.2	

^*a*^ Sens, sensitivity %; Spec, specificity %; CI, 95% confidence interval; mCIM, modified carbapenem inactivation method; mzCIM, modified zinc-supplemented carbapenem inactivation method; sCIM, simplified carbapenem inactivation method.

NitroSpeed-Carba NP, a recently introduced colorimetric assay, offers an intriguing time-to-result of approximately 30 minutes. In its comprehensive version, it enables differentiation between carbapenemase-producing isolates and negative isolates, as well as distinguishing between different Ambler Classes (22, 31, 32). However, the lower performance observed in the current study could be attributed to a significant number of isolates containing weakly carbapenem-hydrolyzing enzymes, such as OXA-23 and OXA-58 in *Proteus* spp., which have not previously been evaluated using this test. In other studies, false-positive results have also been reported, although they could not be linked to the same β-lactamases found in our isolates (22, 33). Nonetheless, it should be noted that we employed a modified protocol for the test, as the original protocol is both labor-intensive and costly, and requires various reagents that are typically not available in the routine microbiology laboratory and are only necessary for the determination of the Ambler Class. Therefore, it is important to consider that the reduction in volume may have influenced the assay’s performance, necessitating further studies to evaluate its efficacy in detecting rare carbapenemases when using the original protocol.

The new CIM-based tests, sCIM and mzCIM, demonstrated excellent performance in detecting both common and rare carbapenemases. This result underscores the potential advantages that these testing approaches could confer in contrast to the presently endorsed methodologies. Further investigations are necessary to confirm this result, ideally using larger isolate collections of different origins. Although sCIM has slightly lower specificity and may produce more indeterminate results compared to mzCIM, it also requires more expertise in handling and coating the antibiotic discs. However, one advantage of sCIM is that it can be set up without the need for prior incubation in broth as for other CIM-based tests. CIM-based tests were also easier to read and less subjective compared to the interpretation of color change with the colorimetric assays.

Our study has some limitations, as the challenge collection included only isolates from Germany. Additionally, some carbapenemases were rare among our collection (GES, GIM), and the performance of the assessed tests might change in countries with a different epidemiology. Nevertheless, our study comprised a large collection of isolates with rare carbapenemases, for which data on the investigated phenotypic detection methods are now available.

### Conclusion

The detection of rare carbapenemases such as OXA-23, GES, or IMI poses a greater challenge compared to common enzymes like KPC, OXA-48, NDM, or VIM. Hydrolysis-based assays have shown promising potential for detecting these enzymes with good sensitivity and specificity. Although colorimetric assays provide faster results (e.g., NitroSpeed-Carba NP in 30 minutes, Carba NP in 120 minutes), it is important to note that their sensitivity for rare carbapenemases is only moderate. On the other hand, both sCIM and the novel mzCIM have demonstrated excellent performance in detecting rare carbapenemase, with a clear gain of sensitivity over the currently recommended mCIM. Additionally, these methods are relatively inexpensive and can be easily implemented in any laboratory setting.

## Data Availability

Molecular characterization of isolates by WGS, including GenBank accession numbers, can be found in Table S2 in the supplemental material.

## SUPPLEMENTAL MATERIAL

Supplementary material spectrum.03015-23-s0001.pdf Tables S1, S2, Figure S1, S2.

ASM does not own the copyrights to Supplemental Material that may be linked to, or accessed through, an article. The authors have granted ASM a non-exclusive, world-wide license to publish the Supplemental Material files. Please contact the corresponding author directly for reuse.
