# Supplementary material for: Detection of rare carbapenemases in Enterobacterales—comparison of two colorimetric and three CIM-based carbapenemase assays
Source: Microbiol Spectr. 2024 Jan 17;12(2):e03015-23. doi: 10.1128/spectrum.03015-23 (PMC10845984; doi:10.1128/spectrum.03015-23)
Supplement: Supplementary material — Tables S1, S2, Figure S1, S2. [file spectrum.03015-23-s0001.pdf]

Detection of rare carbapenemases in Enterobacterales – comparison of two colorimetric and two CIM-based tests

Lukas Schaffarczyk[1], Janina Noster[2], Yvonne Stelzer[2], Janko Sattler[2, 3], Sören Gatermann[4] and Axel Hamprecht[1, 2, 3]

1) University Hospital Oldenburg, Institute for Medical Microbiology and Virology, Oldenburg, Germany
2) Carl von Ossietzky University Oldenburg, Institute for Medical Microbiology and Virology, Oldenburg, Germany
3) Institute for Medical Microbiology, Immunology and Hygiene, University Hospital of Cologne, Cologne, Germany
4) Department of Medical Microbiology, Ruhr University Bochum, Bochum, Germany

Corresponding author
Axel Hamprecht
Institute for medical microbiology and virology
Oldenburg University
axel.hamprecht@uni-oldenburg.de

Supplementary material

Supplementary Table 1 *Carbapenemase producing Enterobacterales (CPE) analysed in this study, subclassified by species and carbapenemase subtype.*

			No. of Isolates with a positive test result[a]				
**β-lactamase type**	**Species**	**β-lactamase**	mCIM	Carba NP	mzCIM	sCIM	NitroSpeed-Carba NP
Carbapenemase producers (n=139)							
Ambler Class A (n = 25)							
	Klebsiella pneumoniae	KPC-2 (3)	3/3 (100)	3/3 (100)	3/3 (100)	3/3 (100)	3/3 (100)
		KPC-3 (2)	2/2 (100)	2/2 (100)	2/2 (100)	2/2 (100)	2/2 (100)
		IMI-2 (1)	1/1 (100)	1/1 (100)	1/1 (100)	1/1 (100)	1/1 (100)
	Klebsiella variicola	KPC-3 (1)	1/1 (100)	1/1 (100)	1/1 (100)	1/1 (100)	1/1 (100)
	Serratia marcescens	KPC-2 (2)	2/2 (100)	2/2 (100)	2/2 (100)	2/2 (100)	2/2 (100)
		GES-5 (3)	**2/3 (67)**	**0/3 (0)**	**2/3 (67)**	2/3 (100)	**2/3 (67)**
	Klebsiella aerogenes	KPC-3 (1)	1/1 (100)	1/1 (100)	1/1 (100)	1/1 (100)	1/1 (100)
	Citrobacter freundii	KPC-3 (2)	2/2 (100)	2/2 (100)	2/2 (100)	2/2 (100)	2/2 (100)
	Citrobacter portucalensis	GES-5 (1)	1/1 (100)	**0/1 (0)**	1/1 (100)	1/1 (100)	1/1 (100)
	Enterobacter cloacae complex	IMI-1 (2)	2/2 (100)	2/2 (100)	2/2 (100)	2/2 (100)	2/2 (100)
		IMI-2 (2)	2/2 (100)	**1/2 (50)**	2/2 (100)	2/2 (100)	**1/2 (50)**
		IMI-3 (1)	1/1 (100)	1/1 (100)	1/1 (100)	1/1 (100)	1/1 (100)
		IMI-4 (1)	1/1 (100)	1/1 (100)	1/1 (100)	1/1 (100)	1/1 (100)
		IMI-9 (1)	1/1 (100)	**0/1 (0)**	1/1 (100)	1/1 (100)	1/1 (100)
		IMI-12 (1)	1/1 (100)	1/1 (100)	1/1 (100)	1/1 (100)	1/1 (100)
		KPC-2 (1)	1/1 (100)	1/1 (100)	1/1 (100)	1/1 (100)	1/1 (100)
Ambler Class B (n = 53)							
	Klebsiella pneumoniae	NDM-1 (2)	**1/2 (50)**	2/2 (100)	2/2 (100)	2/2 (100)	**1/2 (50)**
		NDM-8 (1)	1/1 (100)	1/1 (100)	1/1 (100)	1/1 (100)	1/1 (100)
		NDM-9 (2)	2/2 (100)	2/2 (100)	2/2 (100)	2/2 (100)	2/2 (100)
		VIM-1 (1)	1/1 (100)	1/1 (100)	1/1 (100)	1/1 (100)	1/1 (100)
		VIM-2 (1)	**IR 0/1 (0)**	1/1 (100)	1/1 (100)	1/1 (100)	1/1 (100)
		VIM-19 (1)	1/1 (100)	1/1 (100)	1/1 (100)	1/1 (100)	1/1 (100)
		VIM-46 (1)	**IR 0/1 (0)**	1/1 (100)	1/1 (100)	1/1 (100)	1/1 (100)
		VIM-51 (1)	1/1 (100)	1/1 (100)	1/1 (100)	1/1 (100)	1/1 (100)
		VIM-52 (1)	**IR 0/1 (0)**	1/1 (100)	1/1 (100)	1/1 (100)	1/1 (100)
		IMP-1 (1)	1/1 (100)	1/1 (100)	1/1 (100)	1/1 (100)	1/1 (100)
		IMP-4 (1)	1/1 (100)	1/1 (100)	1/1 (100)	1/1 (100)	1/1 (100)
		IMP-22 (1)	1/1 (100)	1/1 (100)	1/1 (100)	1/1 (100)	1/1 (100)
	Klebsiella oxytoca	VIM-2 (1)	1/1 (100)	1/1 (100)	1/1 (100)	1/1 (100)	1/1 (100)

	Klebsiella pasteurii	VIM-4 (1)	1/1 (100)	1/1 (100)	1/1 (100)	1/1 (100)	1/1 (100)
	Serratia marcescens	VIM-54 (1)	**0/1 (0)**	1/1 (100)	1/1 (100)	1/1 (100)	1/1 (100)
		IMP-13 (2)	**IR 0/2 (0)**	2/2 (100)	2/2 (100)	2/2 (100)	2/2 (100)
		IMP-28 (1)	1/1 (100)	1/1 (100)	1/1 (100)	1/1 (100)	1/1 (100)
		IMP-63 (1)	1/1 (100)	1/1 (100)	1/1 (100)	1/1 (100)	1/1 (100)
	Citrobacter freundii	VIM-1 (1)	1/1 (100)	1/1 (100)	1/1 (100)	1/1 (100)	1/1 (100)
		VIM-2 (2)	**0/2 (0)**	2/2 (100)	2/2 (100)	2/2 (100)	2/2 (100)
		VIM-4 (1)	1/1 (100)	1/1 (100)	1/1 (100)	1/1 (100)	1/1 (100)
		VIM-58 (1)	**0/1 (0)**	**0/1 (0)**	1/1 (100)	1/1 (100)	1/1 (100)
		IMP-1 (1)	1/1 (100)	1/1 (100)	1/1 (100)	1/1 (100)	1/1 (100)
		GIM-1 (2)	**1/2 (50)**	2/2 (100)	2/2 (100)	2/2 (100)	2/2 (100)
	Citrobacter cronae	IMP-8 (1)	1/1 (100)	1/1 (100)	1/1 (100)	1/1 (100)	1/1 (100)
	Citrobacter braakii	NDM-5 (1)	1/1 (100)	1/1 (100)	1/1 (100)	1/1 (100)	1/1 (100)
	Citrobacter portucalensis	VIM-5 (1)	1/1 (100)	1/1 (100)	1/1 (100)	1/1 (100)	1/1 (100)
		VIM-31 (1)	1/1 (100)	1/1 (100)	1/1 (100)	1/1 (100)	1/1 (100)
	Enterobacter cloacae complex	NDM-1 (1)	1/1 (100)	1/1 (100)	1/1 (100)	1/1 (100)	1/1 (100)
		NDM-7 (1)	1/1 (100)	1/1 (100)	1/1 (100)	1/1 (100)	1/1 (100)
		VIM-1 (2)	2/2 (100)	2/2 (100)	2/2 (100)	2/2 (100)	2/2 (100)
		VIM-2 (1)	1/1 (100)	1/1 (100)	1/1 (100)	1/1 (100)	1/1 (100)
		VIM-58 (1)	**IR 0/1 (0)**	1/1 (100)	1/1 (100)	1/1 (100)	1/1 (100)
		GIM-1 (1)	**0/1 (0)**	1/1 (100)	1/1 (100)	1/1 (100)	1/1 (100)
		NDM-5 (1)	1/1 (100)	1/1 (100)	1/1 (100)	1/1 (100)	1/1 (100)
		VIM-4 (1)		1/1 (100)	1/1 (100)	1/1 (100)	1/1 (100)
	Escherichia coli	NDM-3 (1)	1/1 (100)	1/1 (100)	1/1 (100)	1/1 (100)	1/1 (100)
		NDM-4 (2)	2/2 (100)	2/2 (100)	2/2 (100)	2/2 (100)	**1/2 (50)**
		NDM-5 (2)	2/2 (100)	2/2 (100)	2/2 (100)	2/2 (100)	**1/2 (50)**
		VIM-1 (1)	1/1 (100)	1/1 (100)	1/1 (100)	1/1 (100)	1/1 (100)
		IMP-14 (1)	1/1 (100)	1/1 (100)	1/1 (100)	1/1 (100)	1/1 (100)
	Proteus mirabilis	IMP-1 (1)	1/1 (100)	**IR 0/1 (0)**	1/1 (100)	1/1 (100)	1/1 (100)
	Providencia sp.	NDM-1 (1)	1/1 (100)	1/1 (100)	1/1 (100)	1/1 (100)	1/1 (100)
	Providencia stuartii	IMP-1 (1)	1/1 (100)	1/1 (100)	1/1 (100)	1/1 (100)	1/1 (100)
	Raoultella ornithinolytica	NDM-1 (1)	1/1 (100)	1/1 (100)	1/1 (100)	1/1 (100)	1/1 (100)
Ambler Class D (n = 54)							
	Klebsiella aerogenes	OXA-48 (1)	1/1 (100)	1/1 (100)	1/1 (100)	1/1 (100)	1/1 (100)
	Klebsiella pneumoniae	OXA-48 (3)	3/3 (100)	3/3 (100)	3/3 (100)	3/3 (100)	3/3 (100)
		OXA-162 (1)	1/1 (100)	1/1 (100)	1/1 (100)	1/1 (100)	1/1 (100)
		OXA-244 (2)	2/2 (100)	**1/2 (50)**	2/2 (100)	2/2 (100)	2/2 (100)

		OXA-245 (2)	2/2 (100)	2/2 (100)	2/2 (100)	2/2 (100)	2/2 (100)
		OXA-232 (1)	1/1 (100)	1/1 (100)	1/1 (100)	1/1 (100)	1/1 (100)
		OXA-370 (1)	1/1 (100)	1/1 (100)	1/1 (100)	1/1 (100)	1/1 (100)
	Serratia marcescens	OXA-48 (1)	1/1 (100)	1/1 (100)	1/1 (100)	1/1 (100)	1/1 (100)
		OXA-162 (1)	1/1 (100)	1/1 (100)	1/1 (100)	1/1 (100)	1/1 (100)
	Citrobacter freundii	OXA-48 (1)	1/1 (100)	1/1 (100)	1/1 (100)	1/1 (100)	1/1 (100)
		OXA-162 (1)	1/1 (100)	1/1 (100)	1/1 (100)	1/1 (100)	1/1 (100)
	Enterobacter cloacae complex	OXA-48 (1)	1/1 (100)	1/1 (100)	1/1 (100)	1/1 (100)	1/1 (100)
	Escherichia coli	OXA-48 (3)	3/3 (100)	3/3 (100)	3/3 (100)	3/3 (100)	**2/3 (67)**
		OXA-162 (1)	1/1 (100)	1/1 (100)	1/1 (100)	1/1 (100)	1/1 (100)
		OXA-181 (3)	3/3 (100)	3/3 (100)	3/3 (100)	3/3 (100)	3/3 (100)
		OXA-232 (1)	1/1 (100)	1/1 (100)	1/1 (100)	1/1 (100)	1/1 (100)
		OXA-244 (5)	5/5 (100)	**1/5 (20)**	**4/5 (80)**	5/5 (100)	5/5 (100)
	Raoultella ornithinolytica	OXA-48 (1)	1/1 (100)	1/1 (100)	1/1 (100)	1/1 (100)	1/1 (100)
	Proteus mirabilis	OXA-23 (9)	**6/9 (67)**	**0/9 (0)**	9/9 (100)	9/9 (100)	**2/9 (22)**
		OXA-58 (11)	**1/11 (9)**	**1/11 (9)**	**10/11 (91)**	11/11 (100)	**5/11 (46)**
		OXA-162 (1)	1/1 (100)	**IR 0/1 (0)**	1/1 (100)	1/1 (100)	1/1 (100)
		OXA-181 (2)	2/2 (100)	**IR 1/2 (50)**	2/2 (100)	2/2 (100)	2/2 (100)
	Providencia rettgeri	OXA-181 (1)	1/1 (100)	1/1 (100)	1/1 (100)	1/1 (100)	1/1 (100)
Double carbapenemases (n = 7)							
	Klebsiella pneumoniae	NDM-1 + OXA-48 (2)	2/2 (100)	2/2 (100)	2/2 (100)	2/2 (100)	2/2 (100)
		NDM-1 + OXA 232 (1)	1/1 (100)	1/1 (100)	1/1 (100)	1/1 (100)	1/1 (100)
		KPC-2 + OXA-48 (1)	1/1 (100)	1/1 (100)	1/1 (100)	1/1 (100)	1/1 (100)
		NDM-1 + OXA-48 (1)	1/1 (100)	1/1 (100)	1/1 (100)	1/1 (100)	1/1 (100)
	Enterobacter cloacae complex	NDM-1 + OXA-232 (1)	1/1 (100)	1/1 (100)	1/1 (100)	1/1 (100)	1/1 (100)
	Escherichia coli	OXA-181 + NDM-5 (1)	1/1 (100)	1/1 (100)	1/1 (100)	1/1 (100)	1/1 (100)
Non-carbapenemase producers (n = 66)[b]							
	Citrobacter freundii	CTX-M-15 + CMY135 + CMY-2 (1)	0/1 (0)	0/1 (0)	0/1 (0)	0/1 (0)	**FP 1/1 (100)**
		OXA-10 + CTX-M-15 + CMY-135 + CMY-2 (2)	0/2 (0)	0/2 (0)	0/2 (0)	**IR 1/2 (50)**	**FP 2/2 (100)**
		CMY-150 (1)	0/1 (0)	0/1 (0)	0/1 (0)	**FP 1/1 (100)**	**FP 1/1 (100)**
		CMY-152 (1)	0/1 (0)	0/1 (0)	0/1 (0)	0/1 (0)	0/1 (0)
		CMY-86 (1)	0/1 (0)	0/1 (0)	0/1 (0)	0/1 (0)	**FP 1/1 (100)**
	Klebsiella aerogenes	Non (3)	0/3 (0)	0/3 (0)	0/3 (0)	**IR 1/3 (33)**	0/3 (0)
	Klebsiella pneumoniae	OXA-10 (1)	0/1 (0)	0/1 (0)	0/1 (0)	**IR 1/1 (100)**	**FP 1/1 (100)**

	SHV-106 + TEM1-B (1)	0/1 (0)	0/1 (0)	0/1 (0)	0/1 (0)	0/1 (0)
	SHV-108 + TEM1-B (1)	0/1 (0)	0/1 (0)	0/1 (0)	0/1 (0)	0/1 (0)
	OXA-10 + CTX-M-38 (1)	0/1 (0)	0/1 (0)	0/1 (0)	0/1 (0)	0/1 (0)
	CTX-M-1 (1)	0/1 (0)	0/1 (0)	0/1 (0)	0/1 (0)	0/1 (0)
	CTX-M-14 (2)	0/2 (0)	0/2 (0)	0/2 (0)	0/2 (0)	0/2 (0)
	CTX-M-15 (8)	0/8 (0)	0/8 (0)	0/8 (0)	0/8 (0)	0/8 (0)
	DHA-1 (1)	0/1 (0)	0/1 (0)	0/1 (0)	0/1 (0)	0/1 (0)
Klebsiella oxytoca	CTX-M-9 + SHV-12 (1)	0/1 (0)	0/1 (0)	0/1 (0)	0/1 (0)	0/1 (0)
Enterobacter cloacae complex	CTX-M-3 + ACT-7 (1)	0/1 (0)	0/1 (0)	0/1 (0)	**IR 1/1 (100)**	0/1 (0)
	CTX-M-9 + ACT 7 (1)	0/1 (0)	0/1 (0)	0/1 (0)	0/1 (0)	0/1 (0)
	CTX-M-9 + ACT 15 (1)	0/1 (0)	0/1 (0)	0/1 (0)	0/1 (0)	0/1 (0)
	ACT-7 (4)	**FP 1/4 (25)**	0/4 (0)	0/4 (0)	**IR 2/4 (50)**	**FP 2/4 (50)**
	ACT-9 (3)	**IR 1/3 (33)**	0/3 (0)	0/3 (0)	**FP 1/3 (33)**	**FP 1/3 (33)**
	ACT-14 (1)	0/1 (0)	0/1 (0)	0/1 (0)	**IR 1/1 (100)**	**FP 1/1 (100)**
	ACT-15 (1)	0/1 (0)	0/1 (0)	0/1 (0)	0/1 (0)	0/1 (0)
	MIR-5 (1)	0/1 (0)	0/1 (0)	0/1 (0)	0/1 (0)	0/1 (0)
	MIR-6 (1)	0/1 (0)	0/1 (0)	0/1 (0)	**IR 1/1 (100)**	**FP 1/1 (100)**
	Non (1)	0/1 (0)	0/1 (0)	**FP 1/1 (100)**	**FP 1/1 (100)**	**FP 1/1 (100)**
	ACT-16 (2)	0/2 (0)	0/2 (0)	0/2 (0)	**IR 2/2 (100)**	**FP 1/2 (50)**
Serratia marcescens	SRT-2 (2)		0/2 (0)	0/2 (0)	0/2 (0)	**FP 1/2 (50)**
	CTX-M-3 + SRT-2 (1)		0/1 (0)	0/1 (0)	0/1 (0)	0/1 (0)
Escherichia coli	CTX-M-1 (1)	0/1 (0)	0/1 (0)	0/1 (0)	0/1 (0)	0/1 (0)
	CTX-M-3 (1)	0/1 (0)	0/1 (0)	0/1 (0)	0/1 (0)	0/1 (0)
	CTX-M-15 (4)	0/4 (0)	0/4 (0)	0/4 (0)	0/4 (0)	0/4 (0)
	CTX-M-27 (1)	0/1 (0)	0/1 (0)	0/1 (0)	**IR 1/1 (100)**	0/1 (0)
	CTX-M-55 (1)	0/1 (0)	0/1 (0)	0/1 (0)	0/1 (0)	0/1 (0)
	CMY-4 (1)	0/1 (0)	0/1 (0)	0/1 (0)	0/1 (0)	0/1 (0)
Hafnia paralvei	ACC-1 (1)	0/1 (0)	0/1 (0)	0/1 (0)	0/1 (0)	0/1 (0)
	ACC-1a (1)	0/1 (0)	0/1 (0)	0/1 (0)	0/1 (0)	0/1 (0)
Hafnia alvei	ACC-3 (1)	0/1 (0)	0/1 (0)	0/1 (0)	0/1 (0)	0/1 (0)
Proteus mirabilis	none (8)	0/8 (0)	0/8 (0)	0/8 (0)	0/8 (0)	0/8 (0)

Total number = 205

[a] False negative and false-positive results are shown in **bold;** [b] **FP** = false positive, **IR** = indeterminate result

Supplementary Table 2 *Individual results for each isolate tested*

Strain	Species	Accession-Number	Carbapene-mase(s)	Other β-lactamases	MIC (µg/ml)			mCIM result	Carba NP result	mzCIM result	sCIM result	NitroSpeed-Carba NP result
					**EPM**	**IPM**	**MEM**					
**1**	*Enterobacter cloacae complex*	*JAXAEK000000000*	*bla*$_{\text{KPC-2}}$	*bla*$_{\text{ACT-5,}}$ *bla*$_{\text{TEM-1B,}}$ *bla*$_{\text{OXA-1}}$	>32	>32	32	6	pos	6	6	pos
**2**	*Klebsiella pneumoniae*	*JAXAEJ000000000*	*bla*$_{\text{KPC-2}}$	*bla*$_{\text{SHV-106}}$, *bla*$_{\text{OXA-4}}$	8	>32	4	6	pos	6	6	pos
**3**	*Klebsiella pneumoniae*	*JAXAEI000000000*	*bla*$_{\text{KPC-2}}$	*bla*$_{\text{SHV-12}}$, *bla*$_{\text{OXA-9}}$, *bla*$_{\text{TEM-122}}$	>32	>32	>32	6	pos	6	6	pos
**4**	*Klebsiella pneumoniae*	*JAXAEH000000000*	*bla*$_{\text{KPC-2}}$	*bla*$_{\text{CTX-M-15}}$, *bla*$_{\text{SHV-110}}$, *bla*$_{\text{OXA-9}}$, *bla*$_{\text{TEM-1A}}$, *bla*$_{\text{OXA-1}}$	>32	>32	16	6	pos	6	6	pos
**5**	*Serratia marcescens*	*JAXAEG000000000*	*bla*$_{\text{KPC-2}}$	*bla*$_{\text{TEM-1B}}$, *bla*$_{\text{OXA-1}}$	> 8	> 16	> 16	6	pos	6	6	pos
**6**	*Serratia marcescens*	*n.d.*	*bla*$_{\text{KPC-2}}$	*bla*$_{\text{TEM-1B}}$	> 8	> 16	> 16	6	pos	6	6	pos
**7**	*Citrobacter freundii*	*JAXAEE000000000*	*bla*$_{\text{KPC-3}}$	*bla*$_{\text{OXA-10}}$, *bla*$_{\text{CTX-M-15}}$, *bla*$_{\text{CMY-48}}$, *bla*$_{\text{SHV-182}}$, *bla*$_{\text{TEM-1B}}$, *bla*$_{\text{OXA-1}}$	>32	>32	>32	6	pos	6	6	pos
**8**	*Citrobacter freundii*	*JAXAED000000000*	*bla*$_{\text{KPC-3}}$	*bla*$_{\text{CMY-65}}$, *bla*$_{\text{TEM-1A}}$, *bla*$_{\text{OXA-9}}$	2	>32	4	6	pos	6	6	pos
**9**	*Klebsiella aerogenes*	*JAXAEC000000000*	*bla*$_{\text{KPC-3}}$	*bla*$_{\text{TEM-1A}}$, *bla*$_{\text{OXA-9}}$	>32	>32	>32	6	pos	6	6	pos
**10**	*Klebsiella pneumoniae*	*JAXAEB000000000*	*bla*$_{\text{KPC-3}}$	*bla*$_{\text{CTX-M-15}}$, *bla*$_{\text{SHV-106}}$, *bla*$_{\text{OXA-1}}$, *bla*$_{\text{OXA-9}}$	>32	8	>32	6	pos	6	6	pos
**11**	*Klebsiella variicola*	*JAXAEA000000000*	*bla*$_{\text{KPC-3}}$	*bla*$_{\text{LEN17}}$, *bla*$_{\text{TEM-1A}}$, *bla*$_{\text{OXA-9}}$	>32	>32	>32	6	pos	6	6	pos
**12**	*Klebsiella pneumoniae*	*JAXADZ000000000*	*bla*$_{\text{KPC-3}}$	*bla*$_{\text{CTX-M-15}}$, *bla*$_{\text{SHV-182}}$, *bla*$_{\text{TEM-1B}}$, *bla*$_{\text{OXA-1}}$	>32	>32	8	6	pos	6	6	pos
**13**	*Enterobacter cloacae complex*	*JAXAFE000000000*	*bla*$_{\text{IMI-1}}$	*bla*$_{\text{CMH-3}}$	>32	>32	32	6	pos	6	6	pos
**14**	*Enterobacter cloacae complex*	*JAXAFD000000000*	*bla*$_{\text{IMI-1}}$	-	2	>32	1	6	pos	6	6	neg
**15**	*Enterobacter cloacae complex*	*JAXAFC000000000*	*bla*$_{\text{IMI-12}}$	-	>32	>32	>32	6	pos	6	6	pos

**16**	*Enterobacter cloacae complex*	*JAXAFB000000000*	*bla*$_{IMI-2}$	-	>32	>32	>32	6	pos	6	6	pos
**17**	*Enterobacter cloacae complex*	*JAXAFA000000000*	*bla*$_{IMI-2}$	*bla*$_{ACT-9}$	>32	>32	>32	6	neg	6	6	neg
**18**	*Klebsiella pneumoniae*	*JAXAEZ000000000*	*bla*$_{IMI-2}$	*bla*$_{CTX-M-1}$, *bla*$_{SHV-187}$	>32	>32	>32	6	pos	11	8	pos
**19**	*Enterobacter cloacae complex*	*JAXAEY000000000*	*bla*$_{IMI-3}$	*bla*$_{ACT-12}$	8	16	4	6	pos	6	6	neg
**20**	*Enterobacter cloacae complex*	*JAXAEX000000000*	*bla*$_{IMI-4}$	*bla*$_{MIR-2}$	16	>32	16	6	pos	6	6	pos
**21**	*Enterobacter cloacae complex*	*JAXAEW000000000*	*bla*$_{IMI-9}$	-	>32	>32	16	6	neg	6	6	pos
**22**	*Citrobacter portucalensis*	*JAXAFI000000000*	*bla*$_{GES-5}$	*bla*$_{TEM-3}$, *bla*$_{CMY-63}$, *bla*$_{OXA-2}$, *bla*$_{OXA-1}$	>32	>32	>32	6	neg	6	6	pos
**23**	*Serratia marcescens*	*n.d.*	*bla*$_{GES-5}$	-	> 8	8	> 16	27	neg	26	6	neg
**24**	*Serratia marcescens*	*n.d.*	*bla*$_{GES-5}$	-	> 8	8	> 16	27	neg	6	6	neg
**25**	*Serratia marcescens*	*n.d.*	*bla*$_{GES-5}$	-	> 8	> 16	> 16	12+pc	neg	6	6	pos
**26**	*Enterobacter cloacae complex*	*JAXADY000000000*	*bla*$_{NDM-1}$	*bla*$_{ACT-16}$, *bla*$_{OXA-1}$	>32	32	16	6	pos	6	6	pos
**27**	*Klebsiella pneumoniae*	*JAXADX000000000*	*bla*$_{NDM-1}$	*bla*$_{CTX-M-15}$. *bla*$_{SHV-182}$, *bla*$_{OXA-1}$	>32	>32	32	6	pos	6	6	pos
**28**	*Klebsiella pneumoniae*	*JAXADW000000000*	*bla*$_{NDM-1}$	*bla*$_{CTX-M-15}$, *bla*$_{OXA-9}$, *bla*$_{OXA-1}$, *bla*$_{TEM-1B}$, *bla*$_{SHV-106}$	>32	>32	>32	26	pos	6	6	neg
**29**	*Providencia sp*	*JAXADV000000000*	*bla*$_{NDM-1}$	*bla*$_{TEM-1A}$, *bla*$_{VEB-1}$, *bla*$_{OXA-9}$	>32	>32	>32	6	pos	6	6	pos
**30**	*Raoultella ornithinolytica*	*JAXADU000000000*	*bla*$_{NDM-1}$	*bla*$_{NDM-1}$, *bla*$_{CTX-M-15}$. *bla*$_{OXA-1}$	16	8	8	6	pos	6	6	pos
**31**	*Escherichia coli*	*JAXADQ000000000*	*bla*$_{NDM-3}$	*bla*$_{SHV-12}$. *bla*$_{TEM-1B}$	4	4	2	6	pos	6	6	pos
**32**	*Escherichia coli*	*JAXADP000000000*	*bla*$_{NDM-4}$	*bla*$_{OXA-1}$	>32	>32	>32	6	pos	6	6	neg
**33**	*Escherichia coli*	*JAXADO000000000*	*bla*$_{NDM-4}$	*bla*$_{CTX-M-24}$, *bla*$_{CMY-148}$, *bla*$_{TEM-1B}$	32	16	32	6	pos	6	6	pos

**34**	*Citrobacter brakii*	*JAXADN000000000*	*bla*$_{NDM-5}$	*bla*$_{CTX-M-3}$, *bla*$_{CMY-70}$, *bla*$_{TEM-1B}$	32	32	4	6	pos	6	6	pos
**35**	*Enterobacter cloacae complex*	*JAXADM000000000*	*bla*$_{NDM-5}$	*bla*$_{CTX-M-15}$, *bla*$_{ACT-16}$, *bla*$_{TEM-1B}$, *bla*$_{OXA-1}$	>32	>16	>32	6	pos	6	6	pos
**36**	*Escherichia coli*	*JAXADL000000000*	*bla*$_{NDM-5}$	*bla*$_{CMY-59}$, *bla*$_{TEM-1B}$	>32	16	>32	6	pos	6	6	neg
**37**	*Escherichia coli*	*JAXADK000000000*	*bla*$_{NDM-5}$	*bla*$_{CTX-M-15}$, *bla*$_{TEM-1B}$, *bla*$_{OXA-1}$	>32	>32	>32	6	pos	6	6	pos
**38**	*Enterobacter cloacae complex*	*JAXADJ000000000*	*bla*$_{NDM-7}$	*bla*$_{CTX-M-15}$, *bla*$_{ACT-16}$, *bla*$_{OXA-1}$, *bla*$_{TEM-1B}$	>32	>32	>32	6	pos	6	6	pos
**39**	*Klebsiella pneumoniae*	*JAXADI000000000*	*bla*$_{NDM-8}$	*bla*$_{CTX-M-15}$, *bla*$_{SHV-145}$, *bla*$_{TEM-1B}$, *bla*$_{OXA-9}$	>32	32	32	6	pos	6	6	pos
**40**	*Klebsiella pneumoniae*	*JAXADH000000000*	*bla*$_{NDM-9}$	*bla*$_{NDM-9}$, *bla*$_{CTX-M-15}$, *bla*$_{SHV-11}$, *bla*$_{TEM-1A}$, *bla*$_{OXA-9}$	8	8	4	6	pos	6	6	pos
**41**	*Klebsiella pneumoniae*	*JAXADG000000000*	*bla*$_{NDM-9}$	*bla*$_{NDM-9}$, *bla*$_{CTX-M-15}$, *bla*$_{OXA-9}$, *bla*$_{SHV-11}$	8	4	4	6	pos	6	6	pos
**42**	*Citrobacter freundii*	*JAXABZ000000000*	*bla*$_{VIM-1}$	*bla*$_{CMY-109}$	1	4	2	6	pos	6	6	pos
**43**	*Enterobacter cloacae complex*	*JAXABY000000000*	*bla*$_{VIM-1}$	*bla*$_{CTX-M-9}$, *bla*$_{SHV-12}$, *bla*$_{ACT-15}$	1	>32	16	6	pos	6	6	pos
**44**	*Enterobacter cloacae complex*	*JAXABX000000000*	*bla*$_{VIM-1}$	*bla*$_{ACT-7}$	16	32	8	6	pos	6	6	pos
**45**	*Escherichia coli*	*JAXABW000000000*	*bla*$_{VIM-1}$	*bla*$_{TEM-1B}$	1	16	4	6	pos	6	6	pos
**46**	*Klebsiella pneumoniae*	*JAXABV000000000*	*bla*$_{VIM-1}$	*bla*$_{SHV-80}$	0.5	8	4	6	pos	6	6	pos
**47**	*Klebsiella pneumoniae*	*JAXABU000000000*	*bla*$_{VIM-19}$	*bla*$_{CTX-M-15}$, *bla*$_{CMY-4}$, *bla*$_{SHV-145}$	>32	>32	>32	6	pos	6	6	pos
**48**	*Citrobacter freundii*	*JAXABT000000000*	*bla*$_{VIM-2}$	*bla*$_{CMY-152}$, *bla*$_{TEM-1B}$	0.5	8	0.5	26	pos	6	6	pos
**49**	*Citrobacter freundii*	*JAXABS000000000*	*bla*$_{VIM-2}$	*bla*$_{CTX-M-3}$, *bla*$_{CTX-M-9}$, *bla*$_{CMY-152}$, *bla*$_{OXA-129}$	1	15	0.5	25	pos	6	6	pos
**50**	*Enterobacter cloacae complex*	*JAXABR000000000*	*bla*$_{VIM-2}$	*bla*$_{ACT-14}$, *bla*$_{LCR-1}$	>32	8	>32	6	pos	6	6	pos
**51**	*Klebsiella oxytoca*	*JAXABQ000000000*	*bla*$_{VIM-2}$	*bla*$_{OXY-2-2}$	0.06	1	0.125	6	pos	6	6	pos
**52**	*Klebsiella pneumoniae*	*JAXABP000000000*	*bla*$_{VIM-2}$	-	0.125	0.5	0.25	28+pc	pos	6	6	pos

**53**	*Citrobacter portucalensis*	*JAXABO000000000*	*bla*$_{VIM-31}$	*bla*$_{CMY-49}$, *bla*$_{TEM-1B}$	0.5	1	0.5	6	pos	6	6	pos
**54**	*Citrobacter freundii*	*JAXABN000000000*	*bla*$_{VIM-4}$	*bla*$_{CTX-M-15}$, *bla*$_{CMY-65}$, *bla*$_{OXA-1}$	0.25	32	1	6	pos	6	6	pos
**55**	*Enterobacter cloacae complex*	*JAXABM000000000*	*bla*$_{VIM-4}$	*bla*$_{CTX-M-9}$, *bla*$_{ACT-16}$	8	1	1	12+pc	pos	6	6	pos
**56**	*Klebsiella pasteurii*	*JAXABL000000000*	*bla*$_{VIM-4}$	*bla*$_{CTX-M-15}$, *bla*$_{OXY-4-1}$, *bla*$_{OXA-1}$	1	16	2	6	pos	6	6	pos
**57**	*Klebsiella pneumoniae*	*JAXABK000000000*	*bla*$_{VIM-46}$	*bla*$_{CTX-M-15}$, *bla*$_{SHV-106}$, *bla*$_{TEM-1B}$	0.5	4	0.5	25+pc	pos	6	6	pos
**58**	*Citrobacter portucalensis*	*JAXABJ000000000*	*bla*$_{VIM-5}$	*bla*$_{CMY-106}$, *bla*$_{TEM-1B}$, *bla*$_{OXA-2}$	4	4	2	6	pos	6	6	pos
**59**	*Klebsiella pneumoniae*	*JAXABI000000000*	*bla*$_{VIM-51}$	*bla*$_{SHV-1}$	>32	>32	>32	6	pos	6	6	pos
**60**	*Klebsiella pneumoniae*	*JAXABH000000000*	*bla*$_{VIM-52}$	*bla*$_{SHV-11}$	32	>32	8	22+pc	pos	6	6	pos
**61**	*Serratia marcescens*	*JAXABG000000000*	*bla*$_{VIM-54}$	*bla*$_{CTX-M-3}$, *bla*$_{TEM-1B}$	4	>32	>32	28	pos	6	6	pos
**62**	*Citrobacter freundii*	*JAXABF000000000*	*bla*$_{VIM-58}$	*bla*$_{CTX-M-3}$, *bla*$_{CMY-152}$, *bla*$_{TEM-1B}$	1	2	0.25	16+pc	neg	6	6	pos
**63**	*Enterobacter cloacae complex*	*JAXABE000000000*	*bla*$_{VIM-58}$	*bla*$_{CTX-M-9}$, *bla*$_{ACT-15}$, *bla*$_{LAP-2}$	0.5	1	0.125	20+pc	pos	6	6	pos
**64**	*Citrobacter freundii*	*JAXAEV000000000*	*bla*$_{IMP-1}$	*bla*$_{CTX-M-15}$, *bla*$_{OXA-10}$, *bla*$_{CMY-48}$, *bla*$_{OXA-1}$	2	1	2	6	pos	6	6	pos
**65**	*Klebsiella pneumoniae*	*JAXAEU000000000*	*bla*$_{IMP-1}$	*bla*$_{CTX-M-15}$, *bla*$_{SHV-110}$, *bla*$_{TEM-1B}$, *bla*$_{OXA-1}$	>32	8	16	18 + pc	pos	6	6	pos
**66**	*Proteus mirabilis*	*JAPZYJ000000000*	*bla*$_{IMP-1}$	*bla*$_{CTX-M-15}$, *bla*$_{TEM-1B}$, *bla*$_{OXA-1}$,	> 8	> 16	> 16	6	invalid	6	6	pos
**67**	*Providencia stuartii*	*JAXAET000000000*	*bla*$_{IMP-1}$	*bla*$_{CTX-M-15}$, *bla*$_{TEM-1B}$, *bla*$_{OXA-1}$	> 8	> 16	> 16	6	pos	6	6	pos
**68**	*Serratia marcescens*	*JAXAES000000000*	*bla*$_{IMP-13}$	*bla*$_{DHA-1}$, *bla*$_{SRT-2}$, *bla*$_{OXA-1}$	2	4	1	23+pc	pos	6	6	pos
**69**	*Serratia marcescens*	*JAXAER000000000*	*bla*$_{IMP-13}$	*bla*$_{SRT-2}$, *bla*$_{DHA-1}$, *bla*$_{OXA-1}$	4	8	1	25+pc	pos	6	6	pos
**70**	*Escherichia coli*	*JAXAEQ000000000*	*bla*$_{IMP-14}$	-	0.5	0.25	0.5	6	pos	6	6	pos
**71**	*Klebsiella pneumoniae*	*JAXAEP000000000*	*bla*$_{IMP-22}$	*bla*$_{OXA-2}$, *bla*$_{TEM-1A}$, *bla*$_{SHV-106}$, *bla*$_{OXA-9}$	>32	>32	>32	6	pos	6	6	pos
**72**	*Serratia marcescens*	*JAXAEO000000000*	*bla*$_{IMP-28}$	*bla*$_{SRT-2}$	0.25	1	0.25	6	pos	6	6	pos

**73**	*Klebsiella pneumoniae*	*JAXAEN000000000*	*bla*$_{IMP-4}$	*bla*$_{CTX-M-14}$, *bla*$_{DHA-1}$, *bla*$_{TEM-1B}$, *bla*$_{SHV-11}$	4	1	4	6	pos	6	6	pos
**74**	*Serratia marcescens*	*JAXAEM000000000*	*bla*$_{IMP-63}$	-	1	4	0.5	6	pos	6	6	pos
**75**	*Citrobacter cronae*	*JAXAEL000000000*	*bla*$_{IMP-8}$	*bla*$_{OXA-10}$, *bla*$_{CMY-98}$, *bla*$_{OXA-2}$, *bla*$_{OXA-1}$	>32	>32	16	6	pos	6	6	pos
**76**	*Citrobacter freundii*	*JAXAFH000000000*	*bla*$_{GIM-1}$	*bla*$_{CMY-135}$, *bla*$_{CMY-2}$, *bla*$_{OXA-2}$, *bla*$_{TEM-1B}$	8	2	2	27	pos	6	6	pos
**77**	*Citrobacter freundii*	*JAXAFG000000000*	*bla*$_{GIM-1}$	*bla*$_{SHV-12}$, *bla*$_{OXA-10}$, *bla*$_{CMY-152}$, *bla*$_{OXA-2}$, *bla*$_{TEM-2}$	16	8	8	6	pos	6	6	pos
**78**	*Enterobacter cloacae complex*	*JAXAFF000000000*	*bla*$_{GIM-1}$	*bla*$_{ACT-7}$, *bla*$_{OXA-2}$	>32	1	>32	27	pos	6	6	pos
**79**	*Proteus mirabilis*	*JAPZYQ000000000*	*bla*$_{OXA-23}$	-	> 8	> 16	> 16	6	neg	6	6	pos
**80**	*Proteus mirabilis*	*JAPZYF000000000*	*bla*$_{OXA-23}$	-	0.5	> 16	4	6	neg	6	6	neg
**81**	*Proteus mirabilis*	*JAPZYD000000000*	*bla*$_{OXA-23}$	-	1	8	8	6	neg	6	18	neg
**82**	*Proteus mirabilis*	*JAPZYB000000000*	*bla*$_{OXA-23}$	*bla*$_{CMY-138}$, *bla*$_{VEB-6}$, *bla*$_{TEM-1B}$, *bla*$_{TEM-2}$	0.5	16	2	6	invalid	6	6	pos
**83**	*Proteus mirabilis*	*JAPZXX000000000*	*bla*$_{OXA-23}$	-	0.5	> 16	4	16	neg	6	18	neg
**84**	*Proteus mirabilis*	*JAPZXW000000000*	*bla*$_{OXA-23}$	-	0.5	8	1	21	neg	6	18	neg
**85**	*Proteus mirabilis*	*JAPZXV000000000*	*bla*$_{OXA-23}$	-	<0.125	4	0.25	22	neg	6	20	neg
**86**	*Proteus mirabilis*	*JAPZXU000000000*	*bla*$_{OXA-23}$	-	0.5	4	2	6	neg	6	6	neg
**87**	*Proteus mirabilis*	*JAPZXR000000000*	*bla*$_{OXA-23}$	-	0.5	8	1	6	neg	6	6	neg
**88**	*Citrobacter freundii*	*JAXACJ000000000*	*bla*$_{OXA-48}$	*bla*$_{CTX-M-15}$, *bla*$_{CMY-152}$, *bla*$_{TEM-1B}$, *bla*$_{OXA-1}$	4	4	2	6	pos	6	6	pos
**89**	*Enterobacter cloacae complex*	*n.d.*	*bla*$_{OXA-48}$	*bla*$_{CTX-M-15}$, *bla*$_{ACT-7}$, *bla*$_{OXA-1}$, *bla*$_{TEM-1B}$, *bla*$_{LAP-2}$	0.5	2	0.5	6	pos	6	6	pos
**90**	*Escherichia coli*	*JAXACI000000000*	*bla*$_{OXA-48}$	*bla*$_{CTX-M-15}$, *bla*$_{TEM-1B}$	2	0.5	2	6	pos	6	6	neg
**91**	*Escherichia coli*	*JAXACH000000000*	*bla*$_{OXA-48}$	-	>32	1	2	6	pos	6	6	pos

**92**	*Escherichia coli*	*JAXACG000000000*	*bla*$_{\text{OXA-48}}$	*bla*$_{\text{TEM-1B}}$	1	>32	0.5	6	pos	6	6	pos
**93**	*Klebsiella aerogenes*	*JAXACF000000000*	*bla*$_{\text{OXA-48}}$	*bla*$_{\text{CTX-M-15}}$, *bla*$_{\text{OXA-1}}$, *bla*$_{\text{TEM-1B}}$	>32	16	4	6	pos	6	6	pos
**94**	*Klebsiella pneumoniae*	*JAXACE000000000*	*bla*$_{\text{OXA-48}}$	*bla*$_{\text{SHV-145}}$	24	>32	8	6	pos	6	6	pos
**95**	*Klebsiella pneumoniae*	*JAXACD000000000*	*bla*$_{\text{OXA-48}}$	-	2	4	2	6	pos	6	6	pos
**96**	*Klebsiella pneumoniae*	*JAXACC000000000*	*bla*$_{\text{OXA-48}}$	-	32	8	16	6	pos	6	6	pos
**97**	*Raoultella ornithinolytica*	*JAKLSW000000000*	*bla*$_{\text{OXA-48}}$	-	4	>32	1	6	pos	6	6	pos
**98**	*Serratia marcescens*	*JAXACB000000000*	*bla*$_{\text{OXA-48}}$	-	2	>16	0.5	6	pos	6	6	pos
**99**	*Citrobacter freundii*	*JAXADF000000000*	*bla*$_{\text{OXA-162}}$	*bla*$_{\text{CMY-152}}$, *bla*$_{\text{OXA-1}}$, *bla*$_{\text{TEM-1B}}$	4	2	1	6	pos	6	6	pos
**100**	*Escherichia coli*	*JAXADE000000000*	*bla*$_{\text{OXA-162}}$	-	2	1	0.5	6	pos	6	6	pos
**101**	*Klebsiella pneumoniae*	*JAXADD000000000*	*bla*$_{\text{OXA-162}}$	*bla*$_{\text{TEM-1B}}$, *bla*$_{\text{SHV-145}}$	32	8	32	6	pos	6	6	pos
**102**	*Proteus mirabilis*	*JARULR000000000*	*bla*$_{\text{OXA-162}}$	*bla*$_{\text{CTX-M-15}}$	0.5	8	0.5	6	invalid	6	6	pos
**103**	*Serratia marcescens*	*n.d.*	*bla*$_{\text{OXA-162}}$	*bla*$_{\text{SRT-1}}$	> 8	> 16	> 16	6	pos	6	6	pos
**104**	*Escherichia coli*	*JAXADC000000000*	*bla*$_{\text{OXA-181}}$	*bla*$_{\text{CTX-M-15}}$, *bla*$_{\text{OXA-1}}$, *bla*$_{\text{TEM-35}}$	4	0.5	0.25	6	pos	6	6	pos
**105**	*Escherichia coli*	*JAXADB000000000*	*bla*$_{\text{OXA-181}}$	-	2	0.5	0.25	6	pos	6	6	pos
**106**	*Escherichia coli*	*JAXADA000000000*	*bla*$_{\text{OXA-181}}$	-	2	0.25	0.25	6	pos	6	6	pos
**107**	*Proteus mirabilis*	*JAXACZ000000000*	*bla*$_{\text{OXA-181}}$	*bla*$_{\text{TEM-1B}}$, *bla*$_{\text{VEB-6}}$	4	> 16	> 16	6	pos	6	6	pos
**108**	*Proteus mirabilis*	*JAPZXQ000000000*	*bla*$_{\text{OXA-181}}$	-	0.5	8	1	6	invalid	6	6	pos
**109**	*Providencia rettgeri*	*JAXACY000000000*	*bla*$_{\text{OXA-181}}$	*bla*$_{\text{CMY-4}}$	> 8	> 16	> 16	6	pos	6	6	pos
**110**	*Escherichia coli*	*JAXACW000000000*	*bla*$_{\text{OXA-232}}$	*bla*$_{\text{CTX-M-15}}$, *bla*$_{\text{TEM-1B}}$	2	0.5	0.25	6	pos	6	6	pos
**111**	*Klebsiella pneumoniae*	*JAXACV000000000*	*bla*$_{\text{OXA-232}}$	-	>32	>32	>32	6	pos	6	6	pos

**112**	*Escherichia coli*	*JAXACT000000000*	*bla*$_{OXA-244}$	-	8	2	1	6	neg	6	6	pos
**113**	*Escherichia coli*	*JAXACS000000000*	*bla*$_{OXA-244}$	-	8	0.5	2	6	neg	6	6	pos
**114**	*Escherichia coli*	*JAXACR000000000*	*bla*$_{OXA-244}$	-	4	4	1	6	neg	6	6	pos
**115**	*Escherichia coli*	*JAXACQ000000000*	*bla*$_{OXA-244}$	-	2	2	1	6	neg	6	6	pos
**116**	*Escherichia coli*	*JAXACP000000000*	*bla*$_{OXA-244}$	*bla*$_{CTX-M-14b}$, *bla*$_{TEM-1B}$	2	0.5	0.25	6	pos	6	6	pos
**117**	*Klebsiella pneumoniae*	*JAXACO000000000*	*bla*$_{OXA-244}$	-	>32	4	4	6	neg	22	6	pos
**118**	*Klebsiella pneumoniae*	*JAXACN000000000*	*bla*$_{OXA-244}$	*bla*$_{CTX-M-15}$, *bla*$_{OXA-1}$, *bla*$_{SHV-110}$	32	32	32	6	pos	6	6	pos
**119**	*Klebsiella pneumoniae*	*JAXACM000000000*	*bla*$_{OXA-245}$	*bla*$_{CTX-M-15}$, *bla*$_{TEM-1B}$, *bla*$_{SHV-182}$, *bla*$_{OXA-1}$	1	2	2	6	pos	6	6	pos
**120**	*Klebsiella pneumoniae*	*JAXACL000000000*	*bla*$_{OXA-245}$	*bla*$_{SHV-182}$	4	32	32	6	pos	6	6	pos
**121**	*Klebsiella pneumoniae*	*JAXACK000000000*	*bla*$_{OXA-370}$	*bla*$_{CTX-M-15}$, *bla*$_{SHV-182}$	16	4	4	6	pos	6	6	pos
**122**	*Proteus mirabilis*	*JARULS000000000*	*bla*$_{OXA-58}$	*bla*$_{CMY-2}$	<0.125	4	0.25	29	neg	14	6	neg
**123**	*Proteus mirabilis*	*JAPZYP000000000*	*bla*$_{OXA-58}$	-	> 8	> 16	> 16	24	neg	6	6	pos
**124**	*Proteus mirabilis*	*JAPZYO000000000*	*bla*$_{OXA-58}$	-	<0.125	4	< 0.125	27	pos	6	6	pos
**125**	*Proteus mirabilis*	*JAPZYN000000000*	*bla*$_{OXA-58}$	-	4	> 16	> 16	6	neg	6	6	pos
**126**	*Proteus mirabilis*	*JAPZXG000000000*	*bla*$_{OXA-58}$	-	> 8	> 16	> 16	27	neg	6	6	pos
**127**	*Proteus mirabilis*	*JAPZYL000000000*	*bla*$_{OXA-58}$	-	1	8	0.25	28	neg	13	10	pos
**128**	*Proteus mirabilis*	*JAPZYK000000000*	*bla*$_{OXA-58}$	-	> 8	> 16	> 16	30	neg	14	12	pos
**129**	*Proteus mirabilis*	*JAPZYI000000000*	*bla*$_{OXA-58}$	-	< 0.5	4	2	28	neg	23	6	pos
**130**	*Proteus mirabilis*	*JAPZYG000000000*	*bla*$_{OXA-58}$	-	<0.125	8	< 0.25	27	neg	6	16	neg
**131**	*Proteus mirabilis*	*JAPZXP000000000*	*bla*$_{OXA-58}$	-	< 0.5	4	< 0.25	29	neg	13	6	neg

**132**	*Proteus mirabilis*	*JAPZXO000000000*	*bla*$_{\text{OXA-58}}$	-	> 8	>16	8	25	pos	6	6	pos
**133**	*Enterobacter cloacae complex*	*JAXADS000000000*	*bla*$_{\text{NDM-1}}$, *bla*$_{\text{OXA-48}}$	*bla*$_{\text{CTX-M-15}}$, *bla*$_{\text{ACT-16}}$	8	>32	4	6	pos	6	6	pos
**134**	*Klebsiella pneumoniae*	*JAXADR000000000*	*bla*$_{\text{NDM-1}}$, *bla*$_{\text{OXA-48}}$	*bla*$_{\text{CTX-M-15}}$, *bla*$_{\text{SHV-106}}$, *bla*$_{\text{OXA-1}}$, *bla*$_{\text{TEM-1B}}$	>32	>32	>32	6	pos	6	6	pos
**135**	*Klebsiella pneumoniae*	*JAXACA000000000*	*bla*$_{\text{NDM-1}}$, *bla*$_{\text{OXA-48}}$	*bla*$_{\text{CTX-M-15}}$, *bla*$_{\text{SHV-187}}$, *bla*$_{\text{OXA-1}}$, *bla*$_{\text{SHV-187}}$, *bla*$_{\text{TEM-1A}}$	>32	>32	8	6	pos	6	6	pos
**136**	*Klebsiella pneumoniae*	*JAXACU000000000*	*bla*$_{\text{NDM-1,}}$ *bla*$_{\text{OXA-232}}$	-	8	4	2	6	pos	6	6	pos
**137**	*Klebsiella pneumoniae*	*JAXADT000000000*	*bla*$_{\text{NDM-1,}}$ *bla*$_{\text{OXA-232}}$	*bla*$_{\text{CTX-M-15}}$, *bla*$_{\text{CTX-M-15}}$, *bla*$_{\text{OXA-1}}$, *bla*$_{\text{SHV-106}}$, *bla*$_{\text{OXA-1}}$, *bla*$_{\text{TEM-1A}}$	>32	>32	>32	6	pos	6	6	pos
**138**	*Escherichia coli*	*JAXACX000000000*	*bla*$_{\text{NDM-5}}$, *bla*$_{\text{OXA-181}}$	*bla*$_{\text{CTX-M-15}}$, *bla*$_{\text{TEM-1B}}$, *bla*$_{\text{OXA-9}}$	>32	>32	>32	6	pos	6	6	pos
**139**	*Klebsiella pneumoniae*	*JAXAEF000000000*	*bla*$_{\text{KPC-2}}$, *bla*$_{\text{OXA-48}}$	bla$_{\text{CTX-M-3}}$, *bla*$_{\text{SHV-187}}$	>32	32	>32	6	pos	6	6	pos
**140**	*Citrobacter freundii*	JAXABD000000000	neg	*bla*$_{\text{CTX-M-15}}$, *bla*$_{\text{CMY-135}}$, *bla*$_{\text{CMY-2}}$	>32	>32	32	27	neg	30	28	pos
**141**	*Citrobacter freundii*	*JAXABC000000000*	neg	*bla*$_{\text{CMY-150}}$	>32	8	4	28	neg	26	6	pos
**142**	*Citrobacter freundii*	*JAXABB000000000*	neg	*bla*$_{\text{CMY-152}}$	16	4	4	27	neg	28	30	neg
**143**	*Citrobacter portucalensis*	*JAXABA000000000*	Neg	*bla*$_{\text{CMY-86}}$	2	1	0.5	30	neg	27	26	pos
**144**	*Citrobacter freundii*	*JAXAAZ000000000*	neg	*bla*$_{\text{OXA-10}}$, *bla*$_{\text{CTX-M-15}}$, *bla*$_{\text{CMY-135}}$, *bla*$_{\text{CMY-2}}$	>32	>32	8	28	neg	27	29	pos
**145**	*Citrobacter freundii*	*JAXAAY000000000*	neg	*bla*$_{\text{OXA-10}}$, *bla*$_{\text{CTX-M-15}}$, *bla*$_{\text{CMY-135}}$, *bla*$_{\text{CMY-2}}$	>32	>32	8	25	neg	29	25	pos
**146**	*Enterobacter cloacae complex*	*JAXAAX000000000*	neg	*bla*$_{\text{ACT-7}}$	>32	8	4	29	neg	25	30	neg
**147**	*Enterobacter cloacae complex*	*JAXAAW000000000*	neg	*bla*$_{\text{CTX-M-9}}$, *bla*$_{\text{ACT-15}}$	8	1	2	27	neg	29	30	neg
**148**	*Enterobacter cloacae complex*	*JAXAAV000000000*	neg	-	4	4	1	28	neg	6	6	pos
**149**	*Enterobacter cloacae complex*	*JAXAAU000000000*	neg	*bla*$_{\text{ACT-15}}$	4	2	1	27	neg	27	34	neg

**150**	*Enterobacter cloacae complex*	*JAXAAT000000000*	neg	*bla*$_{ACT-7}$	4	1	2	28	neg	28	25	pos
**151**	*Enterobacter cloacae complex*	*JAXAAS000000000*	neg	*bla*$_{ACT-14}$	32	2	16	27	neg	27	23	pos
**152**	*Enterobacter cloacae complex*	*JAXAAR000000000*	neg	*bla*$_{MIR-6}$	1	2	0.25	27	neg	21	24	pos
**153**	*Enterobacter cloacae complex*	*JAXAAQ000000000*	neg	*bla*$_{CTX-M-3}$, *bla*$_{ACT-7}$	16	1	2	28	neg	24	23	neg
**154**	*Enterobacter cloacae complex*	*JAXAAP000000000*	neg	*bla*$_{CTX-M-9}$, *bla*$_{ACT-7}$	4	<0.250	4	27	neg	30	29	neg
**155**	*Enterobacter cloacae complex*	*JAXAAO000000000*	neg	*bla*$_{ACT-9}$	>32	>16	>32	26	neg	21	6	pos
**156**	*Enterobacter cloacae complex*	*JAXAAN000000000*	neg	*bla*$_{MIR-5}$	0.5	1	0.125	28	neg	23	26	neg
**157**	*Enterobacter cloacae complex*	*JAXAAM000000000*	neg	*bla*$_{ACT-7}$	>32	16	8	6	neg	25	29	neg
**158**	*Enterobacter cloacae complex*	*JAXAAL000000000*	neg	*bla*$_{ACT-9}$	8	1	2	27+pc	neg	31	26	neg
**159**	*Enterobacter cloacae complex*	*JAXAAK000000000*	neg	*bla*$_{ACT-7}$	>32	4	4	29	neg	24	24	pos
**160**	*Enterobacter cloacae complex*	*JAXAAJ000000000*	neg	*bla*$_{ACT-9}$	4	1	8	30	neg	24	24	neg
**161**	*Enterobacter cloacae complex*	*JAXAAI000000000*	neg	*bla*$_{ACT-16}$	16	<0.25	2	28	neg	22	23	pos
**162**	*Enterobacter cloacae complex*	*JAXAAH000000000*	neg	*bla*$_{ACT-16}$	>32	>32	>32	30	neg	24	25	neg
**163**	*Escherichia coli*	*JAXAAG000000000*	neg	*bla*$_{CTX-M-55}$	>32	2	8	30	neg	23	29	neg
**164**	*Escherichia coli*	*JAXAAF000000000*	neg	*bla*$_{CTX-M-15}$	8	1	4	28	neg	27	27	neg
**165**	*Escherichia coli*	*JAXAAE000000000*	neg	*bla*$_{CTX-M-15}$	2	<0.25	0.125	27	neg	26	30	neg
**166**	*Escherichia coli*	*JAXAAD000000000*	neg	*bla*$_{CTX-M-15}$	>32	2	8	28	neg	29	28	neg
**167**	*Escherichia coli*	*JAXAAC000000000*	neg	*bla*$_{CMY-4}$	2	<0.25	0.25	28	neg	28	33	neg
**168**	*Escherichia coli*	*JAXAAB000000000*	neg	*bla*$_{CTX-M-1}$	8	2	8	28	neg	28	30	neg
**169**	*Escherichia coli*	*JAXAAA000000000*	neg	*bla*$_{CTX-M-3}$	1	<0.25	0.06	28	neg	27	30	neg

**170**	*Escherichia coli*	*JAWZZZ000000000*	neg	$bla_{\text{CTX-M-27}}$	>32	2	4	27	neg	23	24	neg
**171**	*Escherichia coli*	*JAWZZY000000000*	neg	$bla_{\text{CTX-M-15}}$	32	1	4	27	neg	23	30	neg
**172**	*Hafnia paralvei*	*JAWZZX000000000*	neg	$bla_{\text{ACC-1a}}$	>32	1	8	27	neg	26	28	neg
**173**	*Hafnia alvei*	*JAWZZW000000000*	neg	$bla_{\text{ACC-3}}$	8	1	0.5	28	neg	23	25	neg
**174**	*Hafnia paralvei*	*JAWZZV000000000*	neg	$bla_{\text{ACC-1}}$	>32	8	4	26	neg	30	26	neg
**175**	*Klebsiella aerogenes*	*JAWZZU000000000*	neg	-	>32	4	4	29	neg	26	29	neg
**176**	*Klebsiella aerogenes*	*JAWZZT000000000*	neg	-	>32	>16	4	28	neg	24	28	neg
**177**	*Klebsiella aerogenes*	*JAWZZS000000000*	neg	-	>32	16	8	29	neg	25	22	neg
**178**	*Klebsiella oxytoca*	*JAWZZR000000000*	neg	$bla_{\text{CTX-M-9}}$, $bla_{\text{SHV-12}}$	2	2	2	25	neg	30	28	neg
**179**	*Klebsiella pneumoniae*	*JAWZZQ000000000*	neg	$bla_{\text{CTX-M-14}}$, $bla_{\text{DHA-1}}$	>32	8	4	27	neg	22	28	neg
**180**	*Klebsiella pneumoniae*	*JAWZZP000000000*	neg	$bla_{\text{SHV-76}}$, $bla_{\text{TEM-1B}}$, $bla_{\text{OXA-1}}$	>32	4	8	28	neg	25	29	neg
**181**	*Klebsiella pneumoniae*	*JAWZZO000000000*	neg	$bla_{\text{OXA-10}}$	>32	2	>32	30	neg	21	24	pos
**182**	*Klebsiella pneumoniae*	*JAWZZN000000000*	neg	$bla_{\text{CTX-M-15}}$	8	1	2	26	neg	30	32	neg
**183**	*Klebsiella pneumoniae*	*JAWZZM000000000*	neg	$bla_{\text{DHA-1}}$	>32	16	4	27	neg	28	31	neg
**184**	*Klebsiella pneumoniae*	*JAWZZL000000000*	neg	$bla_{\text{CTX-M-15}}$, $bla_{\text{SHV-27}}$, $bla_{\text{OXA-1}}$	1	1	0.5	30	neg	23	29	neg
**185**	*Klebsiella pneumoniae*	*JAWZZK000000000*	neg	$bla_{\text{CTX-M-1}}$	2	1	1	29	neg	30	30	neg
**186**	*Klebsiella pneumoniae*	*JAWZZJ000000000*	neg	$bla_{\text{CTX-M-14}}$	1	0.25	0.25	27	neg	30	30	neg
**187**	*Klebsiella pneumoniae*	*JAWZZI000000000*	neg	$bla_{\text{SHV-106}}$, $bla_{\text{TEM-1B}}$	>32	16	>32	30	neg	27	28	neg
**188**	*Klebsiella pneumoniae*	*JAWZZH000000000*	neg	$bla_{\text{CTX-M-15}}$, $bla_{\text{TEM-1B}}$, $bla_{\text{SHV-76}}$, $bla_{\text{OXA-1}}$	8	2	2	29	neg	26	28	neg

**189**	*Klebsiella pneumoniae*	*JAWZZG000000000*	neg	*bla*$_{TEM-1B}$, *bla*$_{SHV-110}$	>32	4	6	29	neg	26	27	neg
**190**	*Klebsiella quasipneumoniae*	*JAWZZF000000000*	neg	*bla*$_{OXA-10}$, *bla*$_{CTX-M-38}$	8	0.125	1	28	neg	24	29	neg
**191**	*Klebsiella pneumoniae*	*JAWZZE000000000*	neg	*bla*$_{CTX-M-15}$	8	1	2	26	neg	22	30	neg
**192**	*Klebsiella pneumoniae*	*JAWZZD000000000*	neg	*bla*$_{CTX-M-15}$	8	4	2	30	neg	22	27	neg
**193**	*Klebsiella pneumoniae*	*JAWZZC000000000*	neg	*bla*$_{CTX-M-15}$, *bla*$_{SHV-145}$	8	4	2	30	neg	25	28	neg
**194**	*Klebsiella pneumoniae*	*JAWZZB000000000*	neg	*bla*$_{CTX-M-15}$, *bla*$_{SHV-187}$, blaOXA-1	>32	2	8	29	neg	25	29	neg
**195**	*Proteus mirabilis*	*n.d.*	neg	n.d.	≤0.5	-	≤0.25	29	neg	23	27	neg
**196**	*Proteus mirabilis*	*n.d.*	neg	n.d.	≤0.5	-	≤0.25	28	neg	24	28	neg
**197**	*Proteus mirabilis*	*n.d.*	neg	n.d.	≤0.5	-	≤0.25	28	neg	23	28	neg
**198**	*Proteus mirabilis*	*n.d.*	neg	n.d.	≤0.5	-	≤0.25	26	neg	26	26	neg
**199**	*Proteus mirabilis*	*n.d.*	neg	n.d.	≤0.5	-	≤0.25	28	neg	26	27	neg
**200**	*Proteus mirabilis*	*n.d.*	neg	n.d.	≤0.5	-	≤0.25	27	neg	23	27	neg
**201**	*Proteus mirabilis*	*n.d.*	neg	n.d.	≤0.5	-	≤0.25	28	neg	24	27	neg
**202**	*Proteus mirabilis*	*n.d.*	neg	n.d.	≤0.5	-	≤0.25	28	neg	24	27	neg
**203**	*Serratia marcescens*	*JAWZZA000000000*	neg	*bla*$_{CTX-M-3}$, *bla*$_{SRT-2}$	1	1	0.25	30	neg	29	30	neg
**204**	*Serratia marcescens*	*JAWZYZ000000000*	neg	*bla*$_{SRT-2}$	16	2	2	29	neg	28	26	pos
**205**	*Serratia marcescens*	*JAWZYY000000000*	neg	*bla*$_{SRT-2}$	1	2	0.5	28	neg	30	28	neg

MICs were determined by gradient tests for ertapenem (EPM), imipenem (IPM and meropenem (MEM); mCIM, modified carbapenem inactivation method; mzCIM, modified zinc-supplemented carbapenem inactivation method; sCIM, simplified carbapenem inactivation method; pc, pinpoint colonies; n.d, not determined.

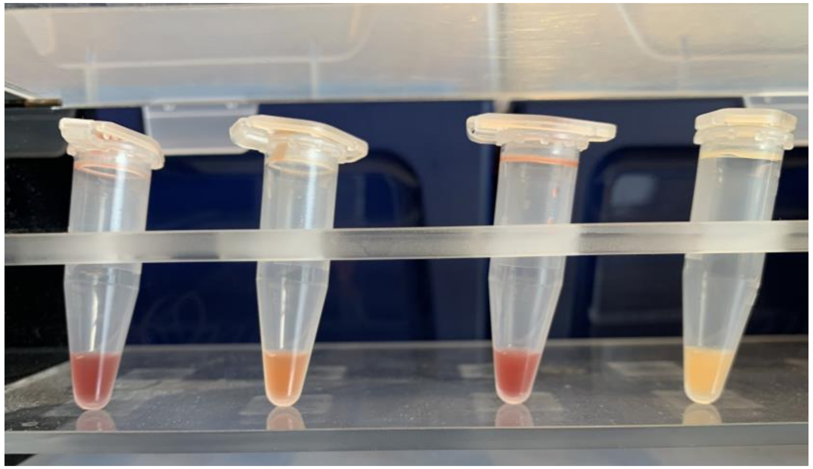

***Supplementary Figure 1** – Examples of NitroSpeed-Carba NP results*

From left to right: positive, positive, positive, negative.

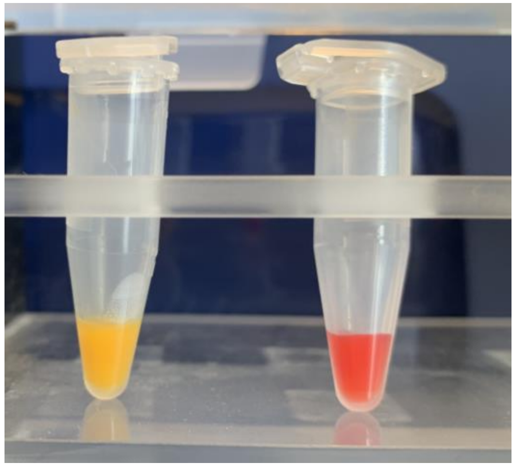

Supplementary Figure 2 *- Examples of Carba NP results*

Left: positive, right negative.
